# Two Doses of Zn Induced Different Microbiota Profiles and Dietary Zinc Supplementation Affects the Intestinal Microbial Profile, Intestinal Microarchitecture and Immune Response in Pigeons

**DOI:** 10.3390/ani14142087

**Published:** 2024-07-17

**Authors:** Dongyan Zhang, Jing Li, Bo Zhang, Yuxin Shao, Zheng Wang

**Affiliations:** Institute of Animal Science and Veterinary Medicine, Beijing Academy of Agriculture and Forestry Sciences, Beijing 100097, China; zhdy203@126.com (D.Z.); xsu1980@163.com (J.L.); zhangb950414@163.com (B.Z.)

**Keywords:** pigeon, Zn, microbiota composition, intestinal morphology, immune indices

## Abstract

**Simple Summary:**

Zinc (Zn) is an essential microelement for normal poultry production. This study determined the microbiota, intestinal morphology and immune status after supplementation with two different doses of Zn in pigeons. The results revealed that Zn can increase the average daily gain and ileal gene expression and improve the serum immune indices and feces microbiota profiles. Our findings provides scientific basis for the application of Zn in pigeon production.

**Abstract:**

We aimed to explore the effects of two different doses of Zn on the fecal microbiota in pigeons and the correlation between these effects and intestinal immune status. Zn doses affected pigeon growth performance, and pigeons in the T60 (60 mg/kg Zn) and T90 (90 mg/kg Zn) groups exhibited higher villus height and crypt depth in duodenum and ileum compared to the control group, respectively. Supplementation with Zn increased the expression of the *IL8*, *CD798*, *TJP* and *NKTR* genes (*p* < 0.05), while enhancing serum immunoglobulin (Ig) G, IgM, and IgA concentrations compared to the control pigeons (*p* < 0.05). T60 treatment reduced relative Actinobacteriota abundance, while *Lactobacillus* spp. abundance was highest in the T90 group compared to the two other groups. The core functional genera significantly associated with immune indices in these pigeons were *Rhodococcus erythropolis* and *Lactobacillus ponti*. Our findings will help facilitate the application of dietary Zn intake in pig production.

## 1. Introduction

The pigeon-breeding industry in China is developing rapidly such that it is the leading global producer of squabs, generating an estimated 700 million per year [1]. Approximately 80% of pigeon meat in the world is produced in China [2], and the number of pigeons bred for consumption is rising by up to 10–15% annually. While nutritional requirements for a range of avian species, including chickens, geese, ducks, and Japanese quails, have been defined by the National Research Council (NRC), no guidelines currently exist for pigeons. Prior studies have explored the requirements of pigeons with respect to energy and crude protein [3] or amino acids [4], but the trace element requirements of these birds remain uncertain. 

Zinc (Zn) is an essential microelement that is required for normal physiological growth, hormone production, muscle development, reproduction, immunity and other processes [5,6,7]. Daily dietary Zn intake is thus vital to ensure the maintenance of appropriate homeostatic balance [8]. The rate of Zn absorption is impacted by the dietary sources of Zn. Poultry feed is generally supplemented with inorganic Zn in the form of ZnSO_4_ and ZnO [9]. No Zn-specific nutritional recommendations have been formulated to date aimed at improving the reproduction and laying performance of breeding pigeons. 

The gastrointestinal microbiota is an integral component of the intestinal microenvironment that plays a critical role in maintaining the health of the host. These gut microbes can help establish a protective barrier layer, regulate metabolic activity, promote immune cell maturation, establish the characteristics of the immune system and influence drug absorption [10,11,12]. Zn can inhibit pathogenic bacterial growth, thereby reducing the risk of bacterial infection [13]. There is also evidence that Zn can influence bacterial persistence and that bacteria use specialized mechanisms to capture Zn in order to regulate their own growth and proliferation. Zn can additionally repress alpha-hemolysin expression, thereby inhibiting the growth of pathogenic *Escherichia coli* [14]. While many studies to date have assessed the importance of Zn in the context of chicken production, there is an absence of any corresponding literature assessing the impact of supplementary Zn intake on the composition of the gastrointestinal microbiome during pigeon production. 

In prior studies, we found that supplemental Zn administration was sufficient to enhance laying performance, sex hormone levels, and eggshell quality in breeding pigeons [15]. In the present analysis, the impacts of dietary Zn administration on growth performance, intestinal morphology, the composition of the gastrointestinal microbiome, and serum immune indices were also assessed in pigeons.

## 2. Materials and Methods

### 2.1. Experimental Design and Dietary Treatments

In total, 72 healthy 1.5-year-old American Silver King pigeon pairs were assigned at random into three treatments with eight replicates and three pens per replicate. According to our previous results, the birds were fed either a control group (basal diet) or the same basal diet supplemented with Zinc heptahydrate (ZnSO4·7H_2_O) at a dose of 60 mg/kg (T60 group) or 90 mg/kg (T90 group), with the final dietary Zn levels being 23.86, 85.69 and 112.14 mg/kg of feed, respectively. Reagent grade Zn sulfate heptahydrate (ZnSO_4_·7H_2_O) was the source of Zn for this study. Individual treatments were comprised of eight replicates (3 cages/replicate), with each 50 × 50 × 60 cm^2^ cage containing one pair of breeding pigeons (1 male, 1 female) and a two-egg clutch. The basal pellet diet fed to these pigeons consisted of a mixture of corn, pea, wheat, soybean meal and sorghum. For details regarding dietary composition and nutrient content, see Table 1. Pigeons had free feed and water access and were maintained in a controlled facility (22 ± 6 °C, 16 h/8 h light/dark cycle). Drinking water contained 0.128 mg/L of Zn and was not considered to be a significant dietary source of this mineral. The total experimental period was 46 days, including 18 days of egg hatching and 28 days of squabs growing.

### 2.2. Sample Collection

At the end of the study, 8 pigeons randomly collected from each group were slaughtered; fresh fecal samples were harvested accordingly; and a total of 24 fecal samples were collected for analyses of microbiota composition. These samples were transferred into sterile containers, snap-frozen using liquid nitrogen and stored at −80 °C. The wing portal venipuncture was additionally used to collect a 5 mL sample of blood prior to morning feeding from 24 pigeons used for fecal sampling. After centrifugation for 10 min at 1238× *g* at 4 °C, serum was collected and stored in the freezer for analyses of immunoglobulin (Ig) G, IgM and IgA concentrations. 

### 2.3. Growth Performance

Pigeons were weighed on days 1 and 28, and the feed intake of each pen was recorded weekly. Using these values, average daily weight gain (ADG) and average daily feed intake (ADFI) were computed.

### 2.4. Intestinal Morphology Analyses

8 pigeons were randomly collected from each group, and duodenal, jejunal, and ileal segments from matching positions were harvested and fixed with 4% paraformaldehyde prior to paraffin embedding and used to prepare 5 mm sections. Following hematoxylin and eosin staining, samples were imaged with a microscope (Olympus CX23, Tokyo, Japan) and analyzed with the ProgRes CapturePro software versions2.8 (Jenoptik, Jena, Germany) to assess intestinal villi height (from the villi tip to crypt opening) and crypt depth (from crypt base to crypt opening).

### 2.5. Ileal Gene Expression Analyses

A qPCR approach was used to measure the mRNA level expression of genes, including interleukin-8 (IL8), occludin (OCLN), CD79b molecule (CD79b), tight junction protein (TJP), zinc finger protein 384 (ZNF384), and natural killer cell triggering receptor (NKTR), using the primers listed in Table 2. Prepared cDNA was diluted two-fold using sterile dH_2_O prior to storage at −20 °C. The PCR settings were as follows: 10 min at 95 °C; 40 cycles of 95 °C for 15 s; appropriate annealing temperatures for 20 s; and 72 °C for 15 s. Gene expression was quantified with a standard curve relative to GAPDH, which served as a reference gene. Sample analyses were conducted in duplicate. 

### 2.6. Analyses of Serum Immune Indices

Serum IgA, IgG and IgM concentrations were measured with commercial ELISA kits (Immunology Consultants Laboratory, Inc., Portland, OR, USA). 

### 2.7. 16S rRNA Sequencing

The E.Z.N.A. Stool DNA kit (Omega Bio-tek, Norcross, GA, USA) was used to extract DNA from fecal samples, after which samples were purified and DNA concentrations were quantified. The bacterial 16S rRNA V3-V4 region was then amplified from these DNA samples via PCR with the following universal primer pair: 338F (5′-ACTC CTACGGGAGGCAGCA-3′) and 806R (5′-GGACTACHVGGGTWTCTAAT-3′). Amplification reactions included 4 μL of 5× FastPfu Buffer, 2 μL of 2.5 mmol/L dNTPs, 0.8 μL of 5 μmol/L F + R primers, 0.4 μL of FastPfu Polymerase, 10 ng of DNA input and ddH_2_O to a final volume of 20 μL. The thermocycler settings were as follows: 95 °C for 3 min; 27 cycles of 95 °C for 30 s, 55 °C for 30 s, and 72 °C for 45 s; and 72 °C for 10 min. The resultant amplicons were separated via 2% agarose gel electrophoresis, extracted, purified and sequenced with the Illumina Miseq PE300 platform (Illumina, San Diego, CA, USA). The composition and alpha diversity of the microbiome was assessed, with samples being distinguished following quality control based on barcode sequences. The Chao1, ACE, Simpson and Shannon indices were used to assess alpha diversity. Taxonomic classification at the 97% operational taxonomic unit (OTU) levels was performed with the RDP classified Bayesian algorithm, and the composition of each sample was analyzed at each taxonomic level. 

### 2.8. Statistical Analyses

All of the data, including ileal gene expression, immune indices, intestinal morphology and alpha diversity indices, were analyzed via one-way ANOVA using the GLM procedure in SAS (SAS Institute, Cary, NC, USA). Differences among means were tested using Tukey’s test, and differences were considered significant at *p* < 0.05. Spearman’s rank correlation analyses were used to assess the associations between microbial taxa and immune index values with JMP v.11 (SAS Institute, Cary, NC, USA). 

## 3. Results

### 3.1. Growth Performance

The impact of dietary Zn levels on ADG and ADFI is presented in Table 3. Pigeons during 1–28 days in the T90 group exhibited higher ADG than those in the control group (*p* = 0.021).

### 3.2. Intestinal Morphology

Significant differences in duodenal villus height, crypt depth and villus: crypt ratios were evident in the T60 and T90 groups compared to the control group, with those pigeons in the T90 group exhibiting the greatest villus height (*p* = 0.048) and a higher villus: crypt ratio (*p* = 0.022) in duodenum compared to the two other groups. In contrast, control pigeons exhibited higher jejunal villi height compared to the T60 and T90 groups (*p* = 0.029). The T60 group also exhibited significantly increased ileal villus height (*p* = 0.038), crypt depth (*p* = 0.047), and villus: crypt ratio values (*p* = 0.044) compared to the pigeons in the two other groups (Table 4).

### 3.3. Ileal Gene Expression Analyses

**Figure 1:**
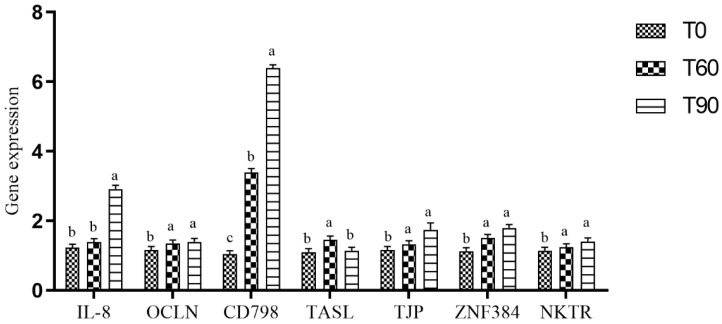
Ileal gene expression analyses in pigeons fed diets containing supplemental Zn. T0: control group; T60: control group + 60 mg/kg Zn; T90: control group + 90 mg/kg Zn. a, b, c significant difference *p* < 0.05.

Increases in *IL8*, *OCLN*, *CD798*, *TJP*, *ZNF384* and *NKTR* expression were evident in the T60 and T90 groups compared to the control group, while significant increases in *IL8*, *CD798*, *TJP* and *ZNF384* expression were evident in the T90 group compared to the two other groups (Figure 1).

### 3.4. Serum Immune Index Analyses

**Figure 2:**
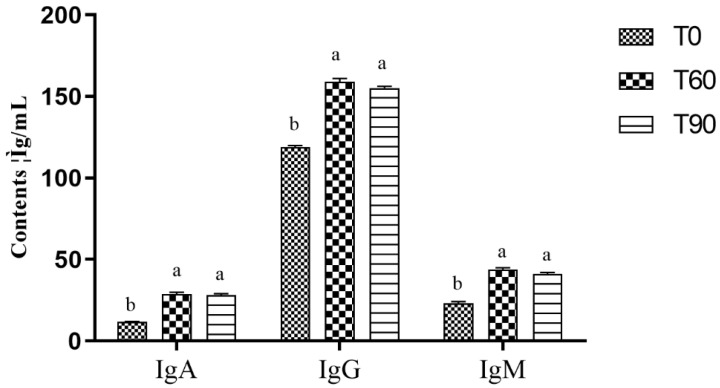
The impact of dietary Zn on pigeon serum immune indices. T0: control group; T60: control group + 60 mg/kg Zn; T90: control group + 90 mg/kg Zn. a, b significant difference *p* < 0.05.

When assessing serum immune indices, significantly higher serum IgG (*p* = 0.034), IgM (*p* = 0.029) and IgA (*p* = 0.042) levels were noted in both the T60 and T90 groups compared to the control group (Figure 2), whereas no differences were noted when comparing the T60 and T90 groups. 

### 3.5. Composition of the Intestinal Microbiome

**Figure 3:**
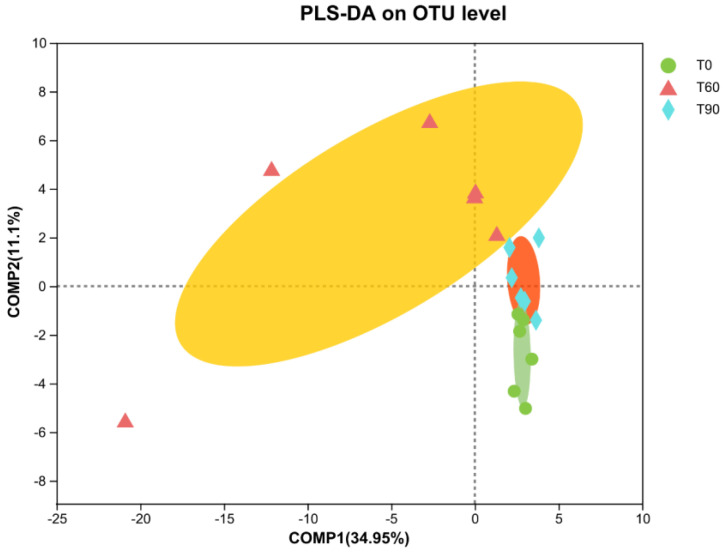
PLS-DA analyses highlighting differences in operational taxonomic unit (OTU) profiles at different taxonomic levels in pigeons from the three experimental groups. T0: control group; T60: control group with 60 mg/kg Zn; T90: control group with 90 mg/kg Zn.

The composition of the intestinal microflora was compared among groups at the OTU level via a PLS-DA approach (Figure 3). This plot revealed clear delineation between the control samples and the two Zn treatment groups with respect to both the bacterial and fungal communities. These analyses demonstrated clear differences in the microbiota profiles in the two doses of Zn treatment groups compared with the control group samples.

No phylum-level differences in Firmicutes abundance were detected among these groups, whereas decreased Actinobacteriota abundance was evident in T60 pigeons compared to the other two groups. 

**Figure 4:**
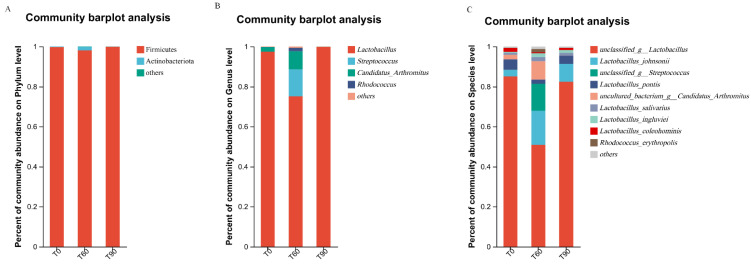
Differences in the composition of the pigeon fecal microbiota among experimental groups. (**A**) phylum level; (**B**) genus levels; (**C**) species level. T0: control group; T60: control group + 60 mg/kg Zn; T90: control group + 90 mg/kg Zn.

When analyzing these samples at the genus level, *Lactobacillus* abundance was, respectively, highest and lowest in the T90 and T60 groups (*p* = 0.013). Significant decreases in *Streptococcus* (*p* = 0.013), *Rhodococcus* (*p* = 0.015), *Candidatus_Arthromitus* (*p* = 0.021), *Enterococcus* (*p* = 0.033) and *Corynebacterium* (*p* = 0.008) abundance were also noted in the T60 group compared to the two other groups. *Lactobacillus pontis* abundance was also increased in the T90 group compared to the controls, whereas *Rhodococcus erythropolis* abundance was enhanced in the T60 group (Figure 4).

### 3.6. The Relationship between Serum Immune Indices, Ileal Gene Expression, and Microbiota Composition

**Figure 5:**
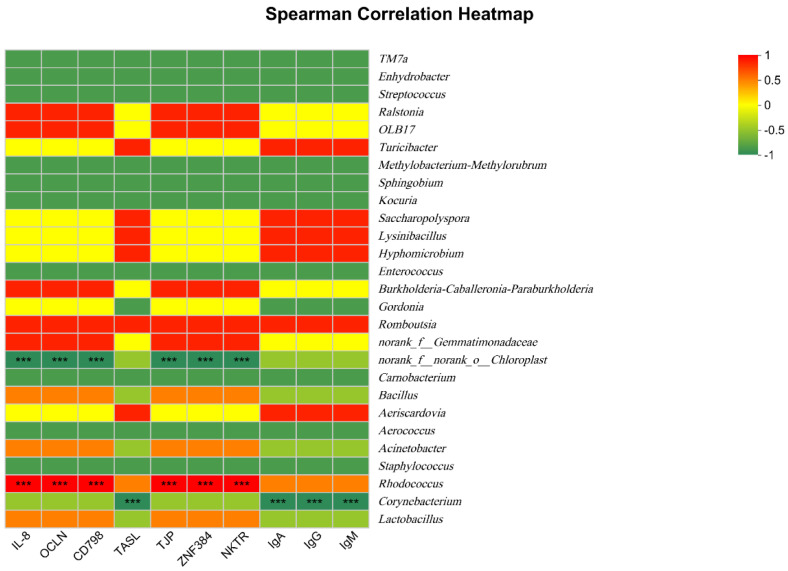
The relationship between serum immune indices, ileal gene expression and microbiota composition. *** significant difference *p* < 0.001.

Lastly, the correlative relationships between microbiota composition and both ileal gene expression and serum immune indices were probed using a Pearson correlation heatmap (Figure 5). The abundance of the genus *Rhodococcus* (Pearson’ *r* = 0.905, *p* = 0.002) was positively correlated with *IL8* expression, while *Romboutsia* abundance was positively correlated with IgA levels (*r* = 0.885, *p* = 0.020). The genera *Enhydrbacter* (*r* = −0.905, *p* = 0.023), *Streptococcus* (*r* = −0.955, *p* = 0.020), *methylobacterium-methylorubrum* (*r* = −0.955, *p* = 0.023), *Sphingobium* (*r* = −0.905, *p* = 0.023), *kocuria* (*r* = −0.855, *p* = 0.023) and *Enterococcus* (*r* = −0.850, *p* = 0.023) were negatively correlated with IgA levels, and Corynebacterium abundance was negatively correlated with levels of IgA (*r* = −0.850, *p* = 0.014), IgG (*r* = −0.754, *p* = 0.022) and IgM (*r* = −0.805, *p* = 0.039).

## 4. Discussion

Despite National Research Council recommendations that chicken diets should ideally include Zn at a concentration of 40 mg/kg (NRC, 1994) [16], broiler breeders supplement Zn administration at doses of 100 mg/kg [17] or 110 mg/kg [18]. Most broiler diets contain Zn levels exceeding reference levels, with some approaching maximum permissible levels (120 mg/kg in the EU) (Additives and Feed, 2014) [19]. This is attributable to the poor Zn utilization exhibited by broilers such that only a small fraction of diet-derived Zn remains in the system [20]. 

Over a 42-day study period, Tang and colleagues [21] observed a dose-dependent increase in ADG and decreased FCR with basal diets supplemented with increasing Zn doses from Zn-bearing clinoptilolite (15.5, 31.0, or 62.0 mg/kg), for Zn concentrations of 86.1 mg/kg and 78.7 mg/kg from days 1–21 and 22–42, respectively. Working with male Arbor Acres broilers Huang and colleagues (2007) [22] additionally tested a range of ZnSO_4_-derived supplemental Zn doses (20, 40, 60, 80, 100, 120, or 140 mg/kg) in a basal diet containing Zn at a dose of 28.37 mg/kg from 1–21 days of age, revealing linear increases in ADG and ADFI without any impact on FCR. These differences may be attributable to variations with respect to the Zn content in the basal diet, levels of supplemental Zn tested, broiler type or growth phase. Humer and colleagues (2015) [23] further determined that Zn exhibited the strongest affinity for phytate, with different basal diet phytate contents potentially explaining varying reports of how supplemental Zn impacts broiler growth performance. In this study, pigeons in the T90 group exhibited higher ADG compared to the control group at study end. Additional research is essential to assess Zn bioavailability in the context of pigeon production. 

The maintenance of an intact mucosal barrier is vital in order to maximize the absorption of nutrients while protecting against intestinal inflammation and associated damage [24,25]. Abnormal gut permeability can permit the passage of pathogens and microbe-derived toxins through the epithelial layer such that they can enter into the vasculature, thereby contributing to intestinal inflammation, reduced performance and an elevated risk of death. As epithelial cells remain in direct contact with the contents of the gastrointestinal lumen, they are highly susceptible to damage such that a reduction in villi is a common hallmark of many intestinal diseases [26]. Conversely, increases in villous surface area are reportedly correlated with improved nutrient uptake [27]. In the present study, significant differences in duodenal villus height, crypt depth and villus: crypt ratio values were evident in the Zn treatment groups compared to the control group. Moreover, the pigeons in the T60 treatment group exhibited increased ileal villus height and villus: crypt ratio values, and the T90 treatment group exhibited increased duodenal villus height and villus: crypt ratio values compared to the control groups. Intestinal permeability is also controlled by a variety of tight junction proteins, including occludins and claudins [28]. Shao and colleagues (2017) [29] previously demonstrated that supplemental dietary Zn intake can enhance the function of the intestinal epithelial barrier as a result of the upregulation of tight junction proteins. The present results also confirmed that higher levels of Zn supplementation were associated with the upregulation of claudin-1 and tight junction protein-1 in the jejunum. Both Zhang and Guo (2009) [30] and Hu and colleagues (2013) [26] also previously reported increases in the expression of these two proteins in response to dietary Zn administration. When claudin-2 is downregulated at the mRNA level, this can contribute to an increase in the permeability of intestinal tight junctions [31]. Compared to the control group in the present study, higher levels of *IL8*, *OCLN*, *CD798*, *TJP*, *ZNF384* and *NKTR* expression were evident in the T60 and T90 groups, with the expression of *IL8*, *CD798*, *TJP* and *ZNF384* also being significantly elevated in the T90 group compared to the T60 and control groups.

The intestinal mucosa serves as a barrier layer and interface between the gastrointestinal immune system and the external environment. As the predominant antibody subtype present within mucosal secretions, IgA is essential for protecting against harmful bacterial entry [32]. Bortoluzzi and colleagues (2019) [33] previously found that providing chickens with supplementary Zn (90 mg/kg) resulted in enhanced jejunal IgA expression following *coccidiosis* challenge with or without *Clostridium perfringens*. The present data revealed that providing pigeons with a 60 mg/kg Zn dose resulted in increases in serum IgA, IgG, and IgM concentrations compared to control pigeons, while no differences in these immunoglobulin concentrations were detected when comparing the T60 and T90 groups.

The gastrointestinal microflora is an essential facet of the intestinal system, playing vital roles in the maintenance of host health through the establishment of a more robust barrier layer, the regulation of immune system maturation, and the control of nutrient and drug metabolic processing and absorption. High-throughput 16S rRNA sequencing analyses provide an effective means of systematically evaluating microbiota composition, thus providing insight into how these microbes shape the health of their hosts. While the use of dietary Zn in the context of animal production is well-established, there has been relatively limited research examining the effects of such supplementation on the intestinal microflora. Zn is an essential mineral that is required for the robust growth of many bacterial species, and Shao and colleagues (2014) [34] previously observed a strong relationship between dietary Zn levels and the composition of the gut microbiome. Yazdankhah and colleagues (2014) [35] additionally noted that Zn exhibits antimicrobial properties with the potential to impact these microbial communities by partially suppressing pathogen growth and reducing fermentation-related nutrient loss. Higher levels of *Lactobacillus* abundance can protect against pathogen colonization via both competitive exclusion and the production of antimicrobial and anti-inflammatory compounds [36]. Shao and colleagues (2014) [33] additionally observed that supplemental Zn intake was beneficial with respect to its effects on *Lactobacillus* abundance, and birds administered Zn at an 80 mg/kg dose previously were found to exhibit higher *Bifidobacterium* and *Enterobacteriaceae* abundance compared to those administered a 100 mg/kg dose [37]. 

Many different studies have similarly found that providing weaning piglets with a diet rich in Zn is associated with increased intestinal *Enterobacteria* abundance and reduced intestinal *Lactobacillus* abundance [38,39,40]. Here, comparisons of changes in the intestinal microbiota in pigeons fed different Zn doses revealed no variations in Firmicutes abundance among these groups, whereas Actinobacteriota abundance was reduced in the T60 group compared to the control and T90 groups. When assessing genus level differences in microbial abundance, the levels of *Lactobacillus* were highest in the T90 group and lowest in the T60 group, while marked reductions in *Streptococcus*, *Rhodococcus*, *Candidatus_Arthromitus*, *Enterococcus* and *Corynebacterium* abundance were also evident in the T60 group compared to the controls. Previous studies demonstrated that a decrease in *Lactobacillus amylovorus* levels in response to high dietary Zn intake [32], whereas effects on other *Lactobacillus* species were less pronounced.

Lastly, a positive correlation was detected between the genus *Rhodococcus* and IL-8 levels, while *Romboutsia* was correlated with IgA concentrations. Conversely, the *Enhydrbacter*, *Streptococcus*, *Sphingobium*, *kocuria* and *Enterococcus* genera were negatively correlated with IgA levels, while *Corynebacterium* was negatively correlated with concentrations of IgA, IgG and IgM. Microbiota analyses further revealed significant increases in *Lactobacillus* abundance in the T90 group, while the abundance of the *Streptococcus*, *Enterococcus* and *Corynebacterium* genera was reduced in the T60 group. Lactic-acid-producing lactobacilli can reportedly lower the gut pH and provide benefits to the digestibility of dietary nutrients [41]. It has been said that the gut microbiota is related to the response of immunity, and beneficial microbiota could improve immunostimulatory or immunosuppressive functions under different health statuses [42]. The present findings suggest that dietary Zn may influence immunological activity in part via regulating *Lactobacillus* abundance, although additional research is vital to better study this relationship. 

## 5. Conclusions

In conclusion, the present study explored the effects of different doses of Zn in pigeons, including growth performance, intestinal morphological characteristics, serum immune indices, ileal gene expression profiles and the composition of the fecal microbiota. While additional research is essential to validate and expand on these findings, they nonetheless provide a valuable foundation that supports the appropriate application of Zn as a means of promoting optimal pigeon production.

## Figures and Tables

**Table 1:** Composition and nutrient levels of the basal diet (air-dry basis).

Ingredients	Content (%)	Nutrient Composition	
Corn	41.00	Metabolizable energy, MJ/kg	11.94
Soybean	13.13	Crude protein ^4^, %	15.91
Pea	17.42	Calcium, %	1.10
Wheat	12.80	Total phosphorus ^4^, %	0.72
Sorghum	10.40	Lys, %	0.88
Soybean oil	0.70	Met, %	0.36
CaHPO_4_·2H_2_O	2.07	Zinc ^4^, mg/kg	23.86
Limestone	1.36		
NaCl	0.40		
Cornstarch + zinc ^1^	0.26		
Vitamin premix ^2^	0.03		
Choline chloride	0.04		
Mineral premix ^3^	0.10		
L-Lysine (98.5%)	0.16		
DL-Methionine	0.13		
Total	100		

^1^ Zinc source replaces equal quality corn starch. ^2^ Complex vitamins are provided per kilogram of feed: Vitamin A 13500IU, Vitamin D_3_ 3600IU, Vitamin E 36IU, Vitamin K_3_ 4.5 mg, Vitamin B_1_ 3.6 mg, Vitamin B_2_ 11.25 mg, D-pantothenic acid 16.5 mg, nicotinamide 39 mg, folic acid 2.1 mg and biotin 0.24 mg. ^3^ Trace components are provided per kilogram of feed: iron 150 mg, copper 8 mg, manganese 65 mg, iodine 0.35 mg, selenium 0.25 mg. ^4^ Analyzed values and each value based on triplicate determinations, and other nutrients were calculated values.

**Table 2:** Primer sequences used for quantitative real-time PCR.

Primer Name	Primer Sequence (5′-3′)
IL-8	F:TTCAGTGGCTGTGTCTCAAGG R:CTTCAACGTTCTTGCAGTGGG
OCLN	F:GTCGCAGTACGAGACCGATT160 R:ACAGAGCTGCTTGTAGCGTT
CD798	F:CTGCCGTTTGCGATGAACAA R:ATTCGCCACCTGTGCTGTTA
TASL	F:GTTCAGAGCGAACATTGCCAG R:GATGAAACAAGTCCCTTTGGGC
TJP	F:TACCACAAGGAGCCATTCCC187 R:GGGTCACAGTGTGGCAGG
ZNF384	F:GAGAGTGAGAGGGGATGGAAGAA R:CAGCCCTTCTCTGTAGGCAATA
NKTR	F:AGAGGATCCAAGCCTTTCCGA R:TTGACAACGGGCACATCTCT

**Table 3:** Effects of dietary zinc level on growth performance of squabs.

Item	T0	T60	T90	*p*-Value	SEM
1~14 d					
ADG (g/d)	26.01	26.72	27.89	0.138	1.730
ADFI (g/d)	114.56	115.07	120.56	0.658	8.242
15~28 d					
ADG (g/d)	9.36	8.47	9.89	0.536	0.584
ADFI (g/d)	175.31	167.79	172.96	0.434	6.342
1~28 d					
ADG (g/d)	17.68 ^b^	17.60 ^b^	18.8 ^a^	0.021	0.851
ADFI (g/d)	144.93	141.43	146.75	0.621	5.212

^a^, ^b^ significant difference *p* < 0.05.

**Table 4:** Intestine morphology in the duodenum, jejunum and ileum of pigeons fed diets supplemented with Zn.

Item	T0	T60	T90	*p*-Value	SEM
Duodenum					
Villus height, μm	769.38 ^b^	682.50 ^c^	802.50 ^a^	0.048	11.049
Crypt depth, μm	28.84 ^b^	41.00 ^a^	34.84 ^b^	0.044	1.460
Villus:Crypt	20.65 ^b^	19.74 ^b^	24.59 ^a^	0.022	1.197
Jejunum					
Villus height, μm	332.69 ^a^	312.99 ^b^	313.57 ^b^	0.029	8.175
Crypt depth, μm	39.20 ^b^	49.00 ^a^	36.78 ^b^	0.008	1.765
Villus:Crypt	1.43 ^a^	1.38 ^a^	1.19 ^b^	0.036	0.044
Ileum					
Villus height, μm	257.59 ^b^	316.94 ^a^	241.48 ^b^	0.078	14.582
Crypt depth, μm	35.30 ^b^	42.24 ^a^	30.38 ^b^	0.047	2.202
Villus:Crypt	7.57 ^b^	7.32 ^b^	8.54 ^a^	0.033	0.125

^a^, ^b^, ^c^ significant difference *p* < 0.05.

## Data Availability

The 16S rRNA data were deposited in NCBI and had been released under “PRJNA991311”.
